# Menaquinone-4 Suppresses Lipopolysaccharide-Induced Inflammation in MG6 Mouse Microglia-Derived Cells by Inhibiting the NF-κB Signaling Pathway

**DOI:** 10.3390/ijms20092317

**Published:** 2019-05-10

**Authors:** Wahyu Dwi Saputra, Nao Aoyama, Michio Komai, Hitoshi Shirakawa

**Affiliations:** 1 Laboratory of Nutrition, Graduate School of Agricultural Science, Tohoku University, 468-1 Aramaki Aza Aoba, Aoba-ku, Sendai 980-8572, Japan; wahyu@g-mail.tohoku-university.jp (W.D.S.); n.aoyama@g-mail.tohoku-university.jp (N.A.); mkomai@m.tohoku.ac.jp (M.K.); 2 International Education and Research Center for Food Agricultural Immunology, Graduate School of Agricultural Science, Tohoku University, 468-1 Aramaki Aza Aoba, Aoba-ku, Sendai 980-8572, Japan

**Keywords:** menaquinone-4, microglia, neuroinflammation, NF-κB

## Abstract

The overactivation of microglia is known to trigger inflammatory reactions in the central nervous system, which ultimately induce neuroinflammatory disorders including Alzheimer’s disease. However, increasing evidence has shown that menaquinone-4 (MK-4), a subtype of vitamin K_2_, can attenuate inflammation in the peripheral system. Whereas it was also observed at high levels within the brain, its function in this organ has not been well characterized. Therefore, we investigated the effect of MK-4 on microglial activation and clarified the underlying mechanism. Mouse microglia-derived MG6 cells were exposed to lipopolysaccharide (LPS) either with or without MK-4 pretreatment. Cell responses with respect to inflammatory cytokines (*Il-1β*, *Tnf-α*, and *Il-6*) were measured by qRT-PCR. We further analyzed the phosphorylation of TAK1, IKKα/β, and p65 of the NF-κB subunit by Western blotting. We observed that in LPS-induced MG6 cells, MK-4 dose-dependently suppressed the upregulation of inflammatory cytokines at the mRNA level. It also significantly decreased the phosphorylation of p65, but did not affect that TAK1 and IKKα/β. Furthermore, the nuclear translocation of NF-κB in LPS-induced MG6 cells was inhibited by MK-4. These results indicate that MK-4 attenuates microglial inflammation by inhibiting NF-κB signaling.

## 1. Introduction

Neuroinflammation has been reported as one of the major causes which initiate neurodegenerative diseases progression including Alzheimer’s Disease (AD) [1,2,3]. It refers to the condition where the persistent stimuli such as age-related oxidative stress and viral infection cause a subsequent activation of microglial cells in the central nervous system (CNS). Microglia represent brain-resident tissue macrophage-like cells that regulate the innate immunity within CNS [4]. After the exposure of several stimuli, microglia release various neurotrophic factors to promote the neuronal cell survival. However, whether the stimulation constantly occurred, microglia will be overactivated and steadily release the proinflammatory and cytotoxicity factors. This aberrant production of proinflammatory mediators further contributes to neuroinflammatory process which is linked to the neuronal degeneration [5,6]. Biesmans et al. reported that lipopolysaccharide (LPS) injection induced the activation of microglia which was followed by the elevated levels of brain IL-1β, IL-6 and TNF-α in mice. Meanwhile, it also caused sickness behavior and reduced the locomotor activity in the aforementioned model [7]. Therefore, the inhibition of microglial overactivation is thought to be a promising therapeutic target to alleviate further neuroinflammation which may cause AD progression.

The activation of microglia by LPS, as shown in Supplementary Figure S1, leads to the Toll-like receptor 4 (TLR4) signal transduction event. This event causes the phosphorylation of IRAK1 which subsequently transmits signal into IKK complex via the activation of TAK1. IKK is the inhibitor of NF-κB kinase which consists of the kinases IKKα and IKKβ. The induction of IKK complex triggers the phosphorylation of IκB (inhibitor of NF-κB) and its degradation by ubiquitin proteasome system allowing the translocation of NF-κB to the nucleus [8]. In addition, several previous studies have reported that MG6 mouse microglia-derived cells can respond to LPS stimulation and actively release inflammatory cytokines [9,10,11]. Moreover, this cell line was also reported to retain several primary microglial cells phenotype characteristic including the morphology, physiology, and immunological activity as well as the microglia-specific substances. Thus, the MG6 cells model can be used as the first door to understand the molecular pathway within the activated microglia [12,13].

Previously known for its role in blood coagulation and bone metabolism, vitamin K was also recognized as a cofactor for the γ-glutamyl carboxylase enzyme. [14]. Naturally, vitamin K is found in two major forms including phylloquinone (vitamin K_1_), which is mostly present in plant-based products, and menaquinone (vitamin K_2_), which is produced by microorganisms including intestinal bacteria [15]. Further, menaquinone-4 (MK-4), a subtype of vitamin K_2_ containing a geranylgeranyl group at the 3-position of 2-methyl-1,4-naphthoquinone, has been reported as the dominant vitamin K form in animal tissues. It was postulated that MK-4 in these tissues originates from the conversion of dietary phylloquinone or is endogenously derived from other vitamin K analogs [16,17,18,19].

Increasing evidence suggests that MK-4 has several unique biological functions such as mediating apoptosis in several cancer cells [20,21,22,23,24,25], promoting growth factor of neuron-like cells [26], enhancing testosterone production [27,28], modulating bile acid synthesis and glucose homeostasis [29], and regulating specific genes via activation of the PXR nuclear receptor [30,31]. Furthermore, vitamin K administration, especially MK-4, was also found to suppress the peripheral inflammation in LPS-induced models [32,33]. Nonetheless, the anti-inflammatory action of MK-4 in activated microglial cells has not been fully illustrated. In addition, whereas MK-4 has been observed at high levels within the brain [16,34], its biological function in this organ has also not been elucidated.

Therefore, in this study, we focused to clarify the effect and the inhibitory mechanism of MK-4 administration against the LPS-induced inflammation in MG6 mouse microglia-derived cells model. Our present study attempted to describe the novel role of MK-4 especially by elucidating its anti-neuroinflammatory action as a scientific basis for further investigation which may contribute to the potential therapeutic strategies to prevent neurodegenerative diseases.

## 2. Results

### 2.1. Effect of MK-4 Pretreatment Time on LPS-Induced Inflammatory Cytokine Expression in MG6 Cells

**Figure 1:**
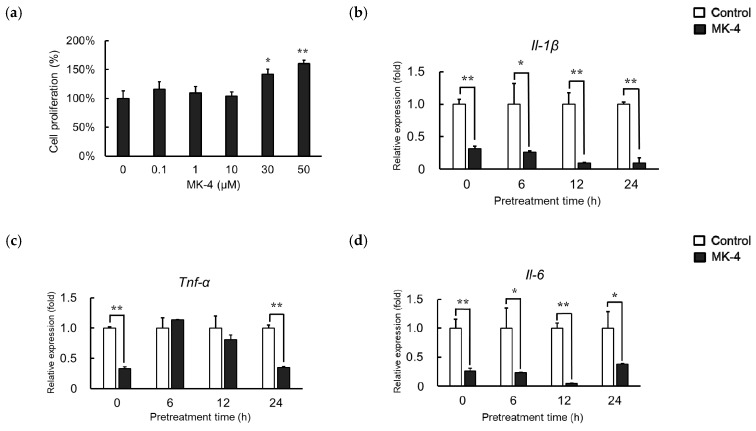
Menaquinone-4 (MK-4) suppresses inflammatory cytokine expression in MG6 cells with different pretreatment times. (**a**) Various doses of MK-4 (0‒50 µM) were administered to MG6 cells for 24 h. Cell viability was measured by WST-1 assays. (**b**,**c**) MG6 cells were pretreated with MK-4 (10 µM) for the indicated times and then exposed with LPS (1 ng/mL) for 3 h. The mRNA levels of proinflammatory cytokines were measured by quantitative reverse transcriptase-PCR, normalized to levels of *Eef1a1* (the internal standard), and expressed as the fold-change relative to control cell values (Cont, cells not treated with MK-4); *Il-1β* (**b**), *Tnf-α* (**c**), and *Il-6* (**d**). Data are presented as the mean ± S.E.M, *n* = 3. Values were significantly different from those of the control at * *p* < 0.05 and ** *p* < 0.01.

We first evaluated whether MK-4 administration affects the MG6 cell viability by performing WST-1 assays. We observed that cell viability was not significantly changed in treatment groups compared to that in the control group at concentrations up to 10 µM (Figure 1a). Therefore, MK-4 concentrations ranging from 0 to 10 µM were used for subsequent experiments. Different pretreatment times were also examined to optimize MK-4 administration. mRNA levels of inflammatory cytokines including *Il-1β*, *Tnf-α*, and *Il-6* were significantly downregulated in the MK-4 group compared to expression in the LPS group, even when MK-4 and LPS were simultaneously administered (Figure 1b–d). Surprisingly, *Tnf-α* level was not significantly different among the treatment groups after 6 and 12 h of administration. Further, prolonged pretreatment of up to 24 h reduced *Tnf-α* levels in the MK-4 group (Figure 1c). Hence, to obtain a stable and optimum MK-4 effect, we used 24 h as the pretreatment time.

### 2.2. Effect of MK-4 on Inflammatory Cytokine and Inflammatory Mediator Expression in LPS-Stimulated MG6 Cells

**Figure 2:**
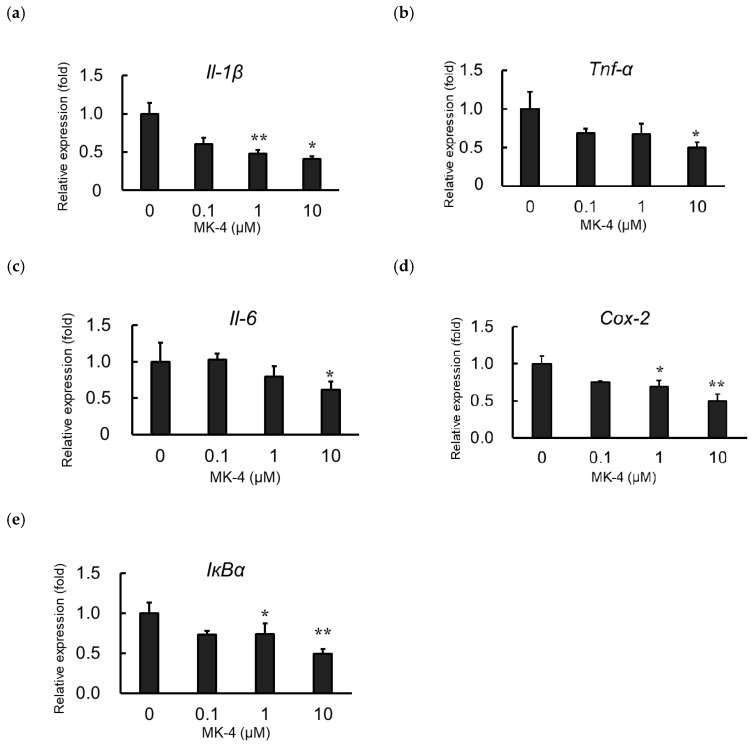
Menaqunine-4 (MK-4) dose-dependently suppresses inflammatory cytokine and inflammatory mediator expression. Various doses of MK-4 (0‒10 µM) were administered to MG6 cells for 24 h, and cells were subsequently exposed to LPS (1 ng/mL) for 3 h. The mRNA levels of inflammatory cytokines and inflammatory mediators were measured by quantitative reverse transcriptase-PCR, normalized to levels of *Eef1a1* (the internal standard), and expressed as the fold-change relative to control cell values (cells not treated with MK-4). (**a**–**c**) The mRNA levels of proinflammatory cytokines *Il-1β* (**a**), *Tnf-α* (**b**), and *Il-6* (**c**). (**d**,**e**) mRNA levels of proinflammatory mediators: *Cox-2* (**d**) and *IκBα* (**e**). Data are presented as the mean ± S.E.M, *n* = 3. Values were significantly different from those of the control at * *p* < 0.05 and ** *p* < 0.01.

To evaluate the effect of MK-4 administration on the expression of inflammatory products, MG6 cells were administered MK-4 for 24 h. Next, cells were stimulated with LPS (1 ng/mL) for 3 h to mimic inflammatory conditions. Inflammatory cytokine mRNA levels, including those of *Il-1β*, *Tnf-α*, and *Il-6*, were markedly suppressed in the MK-4-administered groups, compared to expression in the group without MK-4 administration, after LPS stimulation (Figure 2a–c). MK-4 treatment was also found to reduce the mRNA levels of *Cox-2* and *IκBα* in LPS-induced MG6 cells (Figure 2d,e). These suppressive effects of MK-4 were noted to occur in a dose-dependent manner with the highest suppression at a concentration of 10 µM. Thus, our results indicate that MK-4 can block the upregulation of inflammatory cytokines and inflammatory mediator expression induced by LPS in MG6 cells.

### 2.3. Effect of MK-4 on Inflammatory Cytokine Expression in TNF-α-Stimulated MG6 cells

**Figure 3:**
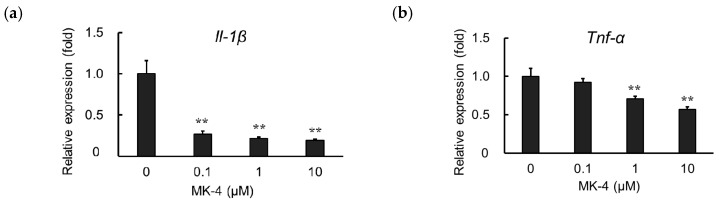
Menaquinone-4 (MK-4) suppresses inflammatory cytokine expression after TNF-α treatment. Various doses of MK-4 (0‒10 µM) were administered to MG6 cells for 24 h, and cells were subsequently exposed to TNF-α (10 ng/mL) for 3 h. The mRNA levels of *Il-1β* and *Tnf-α* were measured by quantitative reverse transcriptase-PCR, normalized to levels of *Eef1a1* (the internal standard), and expressed as the fold-change relative to control cell values (cells not treated with MK-4). (**a**) *Il-1β* levels and (**b**) *Tnf-α* levels. Data are presented as the mean ± S.E.M, *n* = 3. Values were significantly different from those of the control at ** *p* < 0.01.

TNF-α is known to induce inflammatory reactions through its receptors TNFR1 and TNFR2. The interaction between TNF-α and its receptors regulates cellular responses including cell death, survival, proliferation, and migration [35]. However, it was found that macrophage-like cells not only produce TNF-α but are also highly responsive to this cytokine [36]. To evaluate the effect of MK-4 on TNF-α-induced microglial cells, we measured *Il-1β* and *Tnf-α* mRNA expression in MG6 cells after treating them with TNF-α (10 ng/mL) for 3 h. We observed that MK-4 administration also suppressed the upregulation of *Il-1β* and *Tnf-α* in TNF-α-stimulated cells in a dose-dependent manner (Figure 3a,b). These results suggest that MK-4 effectively inhibits inflammatory cytokine production not only in response to LPS stimulation but also in the presence of TNF-α.

### 2.4. Effect of Vitamin K Analogs on Inflammatory Cytokine Expression in LPS-Stimulated MG6 Cells

**Figure 4:**
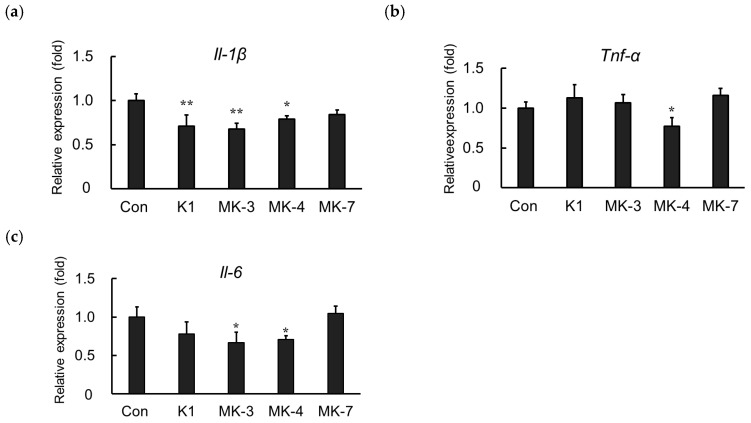
Vitamin K analogs have different effects on inflammatory cytokine expression. MG6 cells were treated with various vitamin K analogs for 24 h and subsequently exposed to LPS (1 ng/mL) for 3 h. The mRNA levels of proinflammatory cytokines were measured by quantitative reverse transcriptase-PCR, normalized to levels of *Eef1a1* (the internal standard), and expressed as the fold-change relative to control cell values (cells not treated with MK-4). (**a**) *Il-1β* levels, (**b**) *Tnf-α* levels, and (**c**) *Il-6* levels. Data are presented as the mean ± S.E.M, *n* = 3. Values were significantly different from those of the control at * *p* < 0.05 and ** *p* < 0.01. K1, phylloquinone (vitamin K_1_); MK-3, menaquinone-3; MK-4, menaquinone-4; MK-7, menaquinone-7.

Here, we showed that MK-4 administration suppresses the mRNA expression of inflammatory cytokines induced by LPS in MG6 cells. We further examined whether other vitamin K analogs including vitamin K_1_, menaquinone-3 (MK-3), and menaquinone-7 (MK-7) could downregulate LPS-induced inflammatory cytokine expression. As shown in Figure 4, LPS-induced *Il-1β* mRNA levels were suppressed not only by MK-4 administration but also by vitamin K_1_ and MK-3 (Figure 4a). Either MK-4 or MK-3 was also able to significantly ameliorate the LPS-induced upregulation of *Il-6* mRNA. However, only MK-4 was found to effectively suppress *Tnf-α* mRNA production in LPS-stimulated MG6 cells (Figure 4c), indicating that it is the optimal vitamin K analog to inhibit inflammation within MG6 cells.

### 2.5. Effect of MK-4 on the Phosphorylation of Inflammation-Related Proteins in LPS-Stimulated MG6 Cells

**Figure 5:**
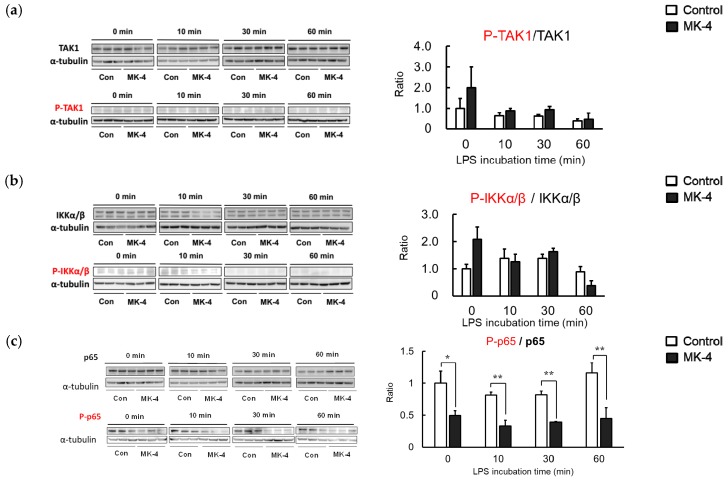
Menaquinone-4 (MK-4) administration reduces phosphorylation of the NF-κB p65 subunit. MG6 cells were treated with MK-4 (10 µM) for 24 h and subsequently exposed to LPS (1 ng/mL). Whole-cell lysates were prepared after 0, 10, 30, and 60 min and analyzed by Western blotting. (**a**–**c**) The protein bands and the ratio of phosphorylated protein was compared to total levels for TAK1 (**a**), IKKα/β (**b**), and p65 (**c**). Data are presented as the mean ± S.E.M, *n* = 3, normalized to α-tubulin levels, and expressed as the fold-change relative to control cell values (con: cells not treated with MK-4 at 0 min post-LPS stimulation). Values were significantly different from those of the control at * *p* < 0.05 and ** *p* < 0.01.

Although we observed that MK-4 administration could inhibit the production of inflammatory factors, the molecular mechanism through which MK-4 suppresses microglial inflammation remained unclear. LPS stimulation induces a phosphorylation cascade that subsequently activates NFκB. To determine the proteins involved in the inhibition of NF-κB by MK-4, we assessed the phosphorylation of inflammation-related proteins in MG6 cells after LPS stimulation. Levels of phosphorylated TAK1 and IKKα/β were not affected by MK-4 after 60 min of LPS stimulation (Figure 5a,b). However, we observed that the phosphorylation of NF-κB p65 was significantly reduced in the MK-4-administered group. The alleviation of NF-κB p65 activation by MK-4 was observed after stimulating the cells with LPS for at least 30 min (Figure 5c). These results indicate that the inhibition of p65 phosphorylation by MK-4 leads to the suppression of NF-κB transcriptional activity in LPS-stimulated MG6 cells.

### 2.6. Effect of MK-4 on the Nuclear Localization of NF-κB p65 in LPS-Stimulated MG6 Cells

**Figure 6:**
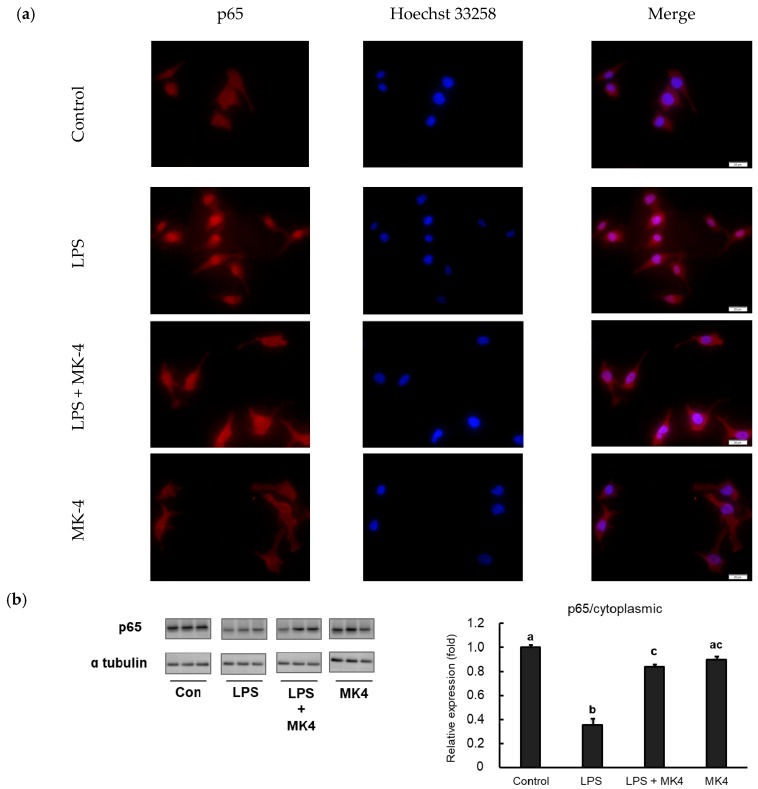

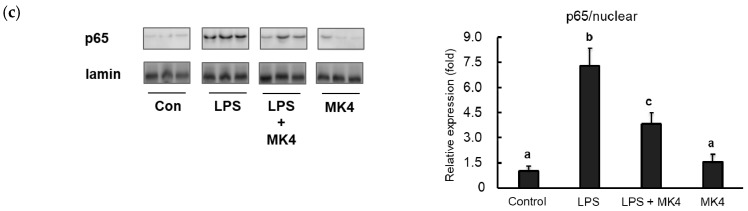
Menaquinone-4 (MK-4) inhibits the LPS-induced nuclear translocation of the NF-κB p65 subunit. (**a**) MG6 cells were administered MK-4 (10 μM) for 24 h and then treated with lipopolysaccharide (1 ng/mL) for 30 min. The cells were fixed and labeled with anti-p65 (red) antibodies. Next, the cells were visualized under a fluorescence microscope. Nuclei were stained with Hoechst 33258 (1 μg/mL) (blue). Scale bars, 20 μm. (**b**–**c**) After MK-4 (10 µM) treatment and LPS (1 ng/mL) administration for 30 min, MG6 cells were fractionated into cytoplasmic and nuclear components. NF-κB was then measured by western blotting in the cytoplasmic (**b**) and nuclear (**c**) fractions. Data are presented as the mean ± SE (*n* = 3), normalized to ɑ tubulin (cytoplasmic) or lamin (nuclear) levels, and expressed as the fold-change relative to control cell values. The values with different letters a–c were significantly different at *p* < 0.05.

Inflammation cascades are triggered by NF-κB when the cells are stimulated with danger molecules including LPS. Under conditions of stimulation, NF-κB translocates from the cytoplasm to the nucleus and subsequently trans-activates the expression of inflammation-related genes. For further analysis, we evaluated the translocation of NF-κB p65 in MG6 cells stimulated with LPS by fluorescence microscopy. p65 was predominantly located in the cytoplasmic area under normal conditions and after MK-4 administration (Figure 6a, first and fourth panels). However, when LPS was added, p65 was found to translocate to the nucleus, indicating that this stimulation induced NF-κB activation (Figure 6a, second panel). In contrast, the administration of MK-4 to LPS-stimulated cells inhibited the nuclear translocation of p65 (Figure 6a, third panel).

To confirm these results, we also measured p65 protein levels in both the cytoplasmic and nuclear fractions of MG6 cells. We observed that LPS stimulation significantly upregulated p65 levels in the nuclear fraction compared to that in control conditions (Figure 6b). In contrast, p65 levels in the LPS-stimulated state were reduced in the cytoplasmic fraction, indicating that p65 translocated to the nucleus (Figure 6c). Consistent with immunofluorescence results, MK-4 administration decreased p65 levels in the nuclear fraction after LPS stimulation (Figure 6b). However, MK-4 itself did not affect p65 levels in either the nuclear (Figure 6b) or cytoplasmic fractions (Figure 6c). Our results suggest that MK-4 administration effectively inhibits the nuclear translocation of p65 in stimulated mouse microglia-derived cells.

## 3. Discussion

Several studies have reported that the overactivation of microglia is strongly related to neuroinflammatory pathologies, which subsequently induce neurodegenerative diseases [37]. Here, we showed that MK-4 administration could manage the inflammatory response in LPS-induced MG6 mouse microglia-derived cells. The pretreatment of MK-4 inhibited the upregulation of proinflammatory cytokines and mediators including *Il-1β*, *Tnf-α*, *Il-6*, *Cox-2*, and *IκBα* due to the LPS stimulation. Meanwhile, it also suppressed the upregulation of *Il-1β* and *Tnf-α* gene transcription after TNF-α stimulation. The other vitamin K analogs also showed this effect even though only in specific genes. Furthermore, MK-4 administration significantly inhibited the phosphorylation and translocation of p65 subunit. These data suggest that the inhibitory action of MK-4 in LPS-induced microglial activation was exerted by modulating NF-κB activation.

The stimulation of LPS and TNF-α has been widely reported to induce the inflammatory cytokines expressions by activating the NF-κB transcription factor. We demonstrated that the different dose of MK-4 treatment reduced these expressions in response to these two stimuli. However, the significant lowering effect after LPS stimulation was acquired only by the administration of 10 µM MK-4. Meanwhile in the TNF-α stimulation, even the lower dose of MK-4 administration also showed the reducing event. While the LPS stimulates NF-κB via the TLR4 pathway, TNF-α induces its activation through the TNFR‒TRAF2 pathway [38,39]. Both pathways converge in the degradation of IκB resulting in a similar change of gene expression. We presume that the difference in response between these two stimuli is due to the difference in the kinetic changes. Magder et al. reported in their experiment that LPS stimulation increased the density of IL-6 later than TNF-α stimulation in endothelial cells [40].

The suppression of inflammatory cytokines expressions by MK-4 has been previously revealed by Ohsaki et al. [33]. They observed that MK-4 could effectively attenuate inflammation in human THP-1 and mouse RAW264.7 macrophage-like cells. In that study, they used a relatively high dose of LPS (1 µg/mL) to induce inflammation within the cells. Interestingly, in our recent study, 1 ng/mL of LPS was sufficient to induce a detectable increase in proinflammatory cytokine expression (Supplementary Figure S2). This phenomenon needs to be clarified; however, we surmise that microglia are probably more responsive to LPS than macrophages.

On the other hand, we found that MK-4 was the optimum vitamin K analogs to manage microglial inflammation compared to phylloquinone (vitamin K_1_) and MK-3. The least effective was exerted by the administration of MK-7 which did not show any significant lowering effect in all measured inflammatory cytokine mRNAs. The similar pattern was also observed in THP-1 macrophage-like cells [33]. Further investigation is necessary to reveal how the isoprenyl side chain of menaquinones modulates the inhibition in the microglial inflammation. However, the majority of vitamin K analog in the CNS was reported to be MK-4 [18,34]. Therefore, our finding would support the hypothesis that MK-4 displays a functional role in this system.

Furthermore, we showed that MK-4 suppressed the inflammation in MG6 cells by measuring the level of NF-κB-related proteins in western blot and fluorescence microscopy. Both these experiments clearly indicated that MK-4 significantly inhibited the phosphorylation and translocation of NF-κB into the nucleus. As LPS stimulation induces the phosphorylation of TAK1 and IKK complex, we did not find any significant difference in these levels between MK-4-untreated and treated groups. We speculate that MK-4 probably does not induce the dissociation of IKK complex, but acts in the downstream level of stimulated MG6 cells to inhibit the activation of NF-κB. Our speculation was due to the phenomenon that in the MK-4-treated groups, the level of IκB was much higher than in the control group in the early time of stimulation (Supplementary Figure S3). This event has also been observed by Yu et al. in rotenone-induced BV2 microglial cells [41]. Rotenone is a widely used pesticide that can transverse cellular membranes and accumulate in the brain to induce dopaminergic neuron impairments. They found that MK-4 could not only inhibit the nuclear localization of NF-κB p65 subunit, but also suppress the phosphorylation of IKKα/β. Compared to our recent data, a different mechanism was found through which MK-4 regulates NF-κB signaling. LPS and rotenone probably exert different inflammation-inducing effects, although this remains unclear. A recent study revealed that LPS exacerbates neurotoxic and inflammatory effects in the substantia nigra when combined with rotenone exposure [42]. The other animal experiment revealed that the rotenone exposure typically does not cause toxic effect in the behaviors of adult rats and induces neurobehavior deterioration only when preceded with LPS exposure [43]. The exact mechanism underlying this phenomenon requires further investigation. However, our data provide evidence that MK-4 could inhibit microglial inflammation not only in response to environmental toxicant exposure but also systemic inflammation.

Currently, the relevance of vitamin K nutritional status to inflammation in humans remains unclear [44]. However, several studies have revealed that high vitamin K levels are associated with lower concentrations of inflammatory markers, conversely, poor vitamin K status was associated with high levels of proinflammatory cytokines [45,46]. It has also been reported that early-stage AD is associated with significantly decreased vitamin K intake [47]. Furtherly, lower vitamin K concentrations were found in the blood of individuals with circulating APOE4, a common genetic marker associated with AD [48]. However, there is still not enough information regarding the correlation between MK-4 intake and the delayed onset of AD pathogenesis. In this experiment, we used MK-4 doses between 0.1 and 10 µM which were in the range of pharmacological dose of MK-4 for human according to the study by Shino et al. [49]. We demonstrated the mechanism and promising results of MK-4 administration effect in the cell-based experiment. Therefore, a further in vivo experiment should be performed in order to confirm these results and elucidate the detailed mechanisms.

In summary, we observed that MK-4 administration could effectively suppress the inflammation in LPS-induced MG6 mouse microglia-derived cells. The inhibitory action of MK-4 was exerted via the suppression of the inflammatory cytokines production possibly by inhibiting the phosphorylation and nuclear localization of the NF-κB. Since it has been previously reported that MK-4 is contained in relatively high amount in the extrahepatic tissues, our findings provide a novel clue for further analysis regarding the role of MK-4 in these organs especially in the CNS.

## 4. Materials and Methods

### 4.1. Materials

MK-4 was kindly provided by Nisshin Pharma Inc. (Tokyo, Japan); menaquinone-3 and menaquinone-7 were obtained from Eisai (Tokyo, Japan) and J-Oil Mills (Tokyo, Japan), respectively. Ethanol was purchased from Wako Pure Chemicals (Osaka, Japan) and used to dissolve all vitamin K analogs at a concentration of 10 mM. The concentration of ethanol as a solvent was adjusted to 0.1% when vitamin K was added to the cell culture medium. 

### 4.2. Cell Culture

c-*Myc*-immortalized MG6 mouse microglial cells [12,13] (RCB2403, RIKEN Cell Bank, Tsukuba, Japan) were seeded in 10-cm Petri dishes and maintained in DMEM (Sigma Aldrich) containing 10% of fetal bovine serum (Biosera, Nuaillè, France), 100 µM β-mercaptoethanol (Wako, Osaka, Japan), 10 µg/mL human recombinant insulin (Gibco^TM^ Thermo Fisher Scientific), 100 µg/mL streptomycin, and 100 U/mL penicillin at 37 °C with an atmosphere of 5% CO_2_. Cell growth and morphology were monitored under an inverted microscope and cells were passaged when they reached a minimum confluency of 80%.

### 4.3. WST-1 Assays

MG6 cells were seeded in 96-well plates at a density of 1.0 × 10^4^ cells/well for overnight culture. On the following day, the culture medium was changed to fresh medium containing MK-4 at the indicated concentrations. Ethanol was dissolved in the medium at a ratio of 1% (*v*/*v*) and used as the control group. After 24-h incubation, WST-1 reagent was added to every well and the absorbances (450 nm) were measured using a microplate reader 680 XR (BIO-RAD) after 0.5, 1, 2, 3, and 4 h of incubation. The three most stable values among readings were used to calculate the cell viabilities.

### 4.4. RNA Extraction and Quantitative Reverse Transcriptase Polymerase Chain Reaction 

MG6 cells were seeded in 6-cm dishes at a density of 1.5 × 10^6^ cell/mL and incubated with culture medium containing MK-4 or other vitamin K analogs at the indicated concentrations and for the indicated times. Next, LPS (1 ng/mL, *Escherichia coli* serotype O111: B4, L2630: Sigma) was added for 3 h. Total RNA was extracted and isolated using ISOGEN reagent (Nippon Gene, Tokyo, Japan). To determine the RNA quantity and quality, the absorbance of isolated RNA was assessed spectrophotometrically at 260 nm in relation to that at 280 nm. cDNA synthesis was performed by denaturing the RNA with 2.5 µM oligo-dT primer (Hokkaido System Science Co., Sapporo, Japan) and 0.5 mM dNTP (GE Healthcare, Tokyo, Japan) for 5 min at 65 °C. RNA was then incubated with the RT buffer (50 mM tris-HCl (pH 8.3), 75 mM KCl, 3 mM MgCl_2_, and 5 mM dithiothreitol) containing 50 U SuperScript III reverse transcriptase (Invitrogen, Carlsbad, CA, USA) and 20 U RNaseOUT RNase inhibitor (Invitrogen) at 50 °C for 60 min for reverse transcription. An aliquot of cDNA was used as a template for quantitative RT-PCR using the Applied Biosystems 7300 Real-Time PCR System (Foster City, CA, USA). The gene-specific primers (Table 1) and SYBR Premix Ex Taq solution (Takara Bio, Otsu, Japan) were used to amplify the target cDNAs. The mRNA levels were then normalized to the levels of eukaryotic elongation factor 1α1 (*Eef1a1*) as the internal standard.

### 4.5. Western Blot Analysis

MG6 cells were seeded in 10-cm dishes at 6 × 10^6^ cells/dish. The following day, MK-4 was administered for 24 h. Next, LPS (1 ng/mL) was added for the indicated times. Cells then were harvested by discarding the medium and scraping the cells in cold 1× PBS. After removing PBS by centrifugation, pellets were mixed with whole cell lysis buffers containing 50 mM tris-HCl (pH 7.5), 150 mM NaCl, 5 mM EDTA, 0.1% SDS, and a proteinase and phosphatase inhibitor cocktail (Roche Applied Science) for 30 min on ice. The lysate was collected after removing the debris by centrifugation at 10,000× *g* at 4 °C for 15 min. For the fractionation assay, cells were first lysed in cytoplasmic lysis buffer containing 320 mM sucrose, 3 mM CaCl_2_, 0.1 mM EDTA, 1 mM DTT, 2 mM MgCl_2_, 0.5% NP-40, and inhibitor cocktails for 20 min in ice. After centrifugation at 10,000× *g* at 4 °C for 10 min, the supernatant was collected as the cytoplasmic fraction and pellet were washed with cytoplasmic buffer without NP-40. The pellet was then lysed with nuclear lysis buffer containing 20 mM HEPES (pH 7.9), 25% glycerol, 1 mM DTT, 1.5 mM MgCl_2_, 280 mM KCl, 0.2 mM EDTA, 0.3% NP-40, and inhibitor cocktails and sonicated. Next, the debris was removed by centrifugation, as with the previous conditions, and supernatants were collected as the nuclear fraction. The protein concentration was determined using the Lowry method and mixed with SDS gel loading buffer. Samples were resolved by 10–20% SDS–polyacrylamide gel electrophoresis (Wako Pure Chemicals) and transferred to polyvinylidene fluoride membranes (Millipore, Billerica, MA, USA). The membranes were incubated with antibodies against TAK1 (Cell Signalling Technology, Danvers, MA, USA), IKKα (Cell Signalling Technology), IKKβ (Cell Signalling Technology), p65 (Cell Signalling Technology), IκBα (Cell Signalling Technology), phospho-TAK1 (Thr184/187; Cell Signalling Technology), phospho-IKKα/β (Ser176/180; Cell Signalling Technology), or phospho-p65 (Ser536; Cell Signalling Technology). All the primary antibodies were diluted in the blocking buffer at the dilution of 1:1000. The bands were detected with the Immobilon Western Detection Reagent (Millipore) using the luminescent image analyzer LAS-4000 mini (Fujifilm, Tokyo, Japan). The relative expression levels of each protein were normalized to the level of α-tubulin (1:5000) (Sigma) for whole lysate samples and cytoplasmic fractions or lamin A/B (1:3000) (Cell Signalling Technology) for nuclear fractions.

### 4.6. Fluorescence Microscopy

MG6 cells were plated in four-chamber plates (Nunclon Thermo Scientific) at a density of 5 × 10^4^ cells/chamber overnight. The following day, the medium was changed to fresh medium containing treatment reagents. After 24 h, LPS (1 ng/mL) was added for 30 min, and then the medium was discarded. Cells were washed with cold 1× PBS and subsequently fixed in 4% formaldehyde/PBS for 15 min. The fixative solution was aspirated, which was followed by permeabilization using 0.25% triton X-100/PBS for 5 min. Cells were then blocked for 1 h at room temperature using 4% fetal bovine serum (Gibco) in PBS buffer containing 0.05% tween-20. An anti-p65 (1:100) (Cell Signalling Technology) antibody was used as the primary antibody, and cells were incubated overnight at 4 °C, which was followed by incubation with the Alexa Fluor 555 goat anti-rabbit (1:200) (Invitrogen) for 1 h at room temperature. Nuclear staining was carried out by incubating cells with Hoechst 33258 (1:10,000) (Dojindo, Kumamoto, Japan) for 10 min at room temperature.

### 4.7. Statistical Analysis

The results are presented as the mean ± SEM (standard error of mean). The data were analyzed by performing a one-way analysis of variance (ANOVA), except for those of Figure 6b,c, which were analyzed using a two-way ANOVA. Multiple comparisons were tested using the Student’s t-test, Dunnett’s test, or Tukey–Kramer test in SigmaPlot version 12.5 (San Jose, CA, USA). Statistical analysis was performed at the significance levels indicated in each figure. 

## Figures and Tables

**Table 1:** Nucleotide sequences of primers used for quantitative RT-PCR.

Gene	Forward Primer	Reverse Primer
*Il-1β*	CTGTGTCTTTCCCGTGGACC	CAGCTCATATGGGTCCGACA
*Tnf-α*	GACGTGGAACTGGCAGAAGAG	TCTGGAAGCCCCCCATCT
*Il-6*	AGAGGAGACTTCACAGAGGATACC	AATCAGAATTGCCATTGCACAAC
*Cox-2*	TGAGTACCGCAAACGCTTCT	CAGCCATTTCCTTCTCTCCTGT
*IκBα*	CTTGGGTGCTGATGTCAATG	ACCAGGTCAGGATTTTGCAG
*Eef1a1*	GATGGCCCCAAATTCTTGAAG	GGACCATGTCAACAATTGCAG

## Supplementary Materials

The supplementary materials are available online at https://www.mdpi.com/1422-0067/20/9/2317/s1.

## Abbreviations

AD	Alzheimer’s disease
CNS	Central nervous system
LPS	Lipopolysaccharide
MK-3	Menaquinone-3
MK-4	Menaquinone-4
MK-7	Menaquinone-7
