# Supplementary figures and images for: Menaquinone-4 Suppresses Lipopolysaccharide-Induced Inflammation in MG6 Mouse Microglia-Derived Cells by Inhibiting the NF-κB Signaling Pathway
Source: Int J Mol Sci. 2019 May 10;20(9):2317. doi: 10.3390/ijms20092317 (PMC6540242; doi:10.3390/ijms20092317)

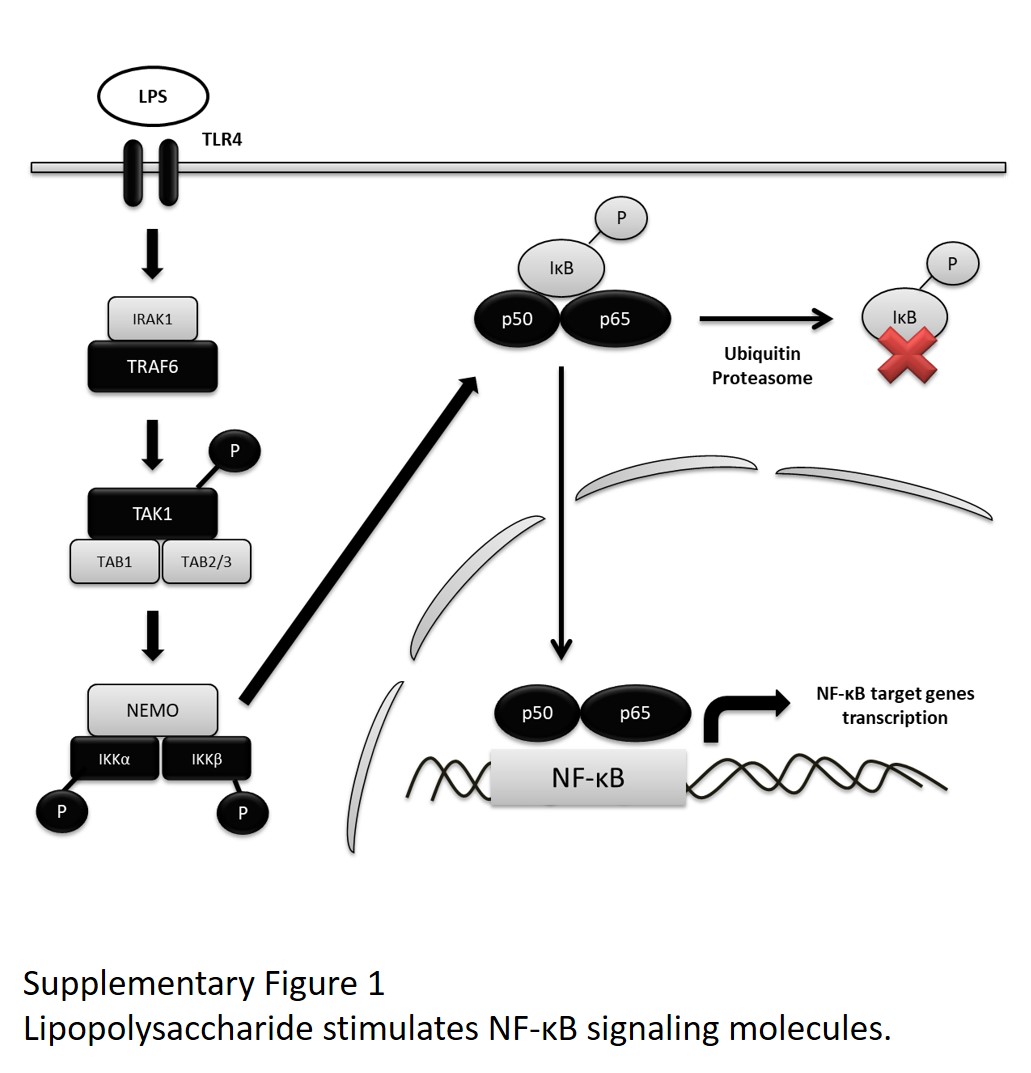

Supplement: Supplementary file 1 [file ijms-20-02317-s001.zip › Sup figures/Sup Fig 1.JPG]

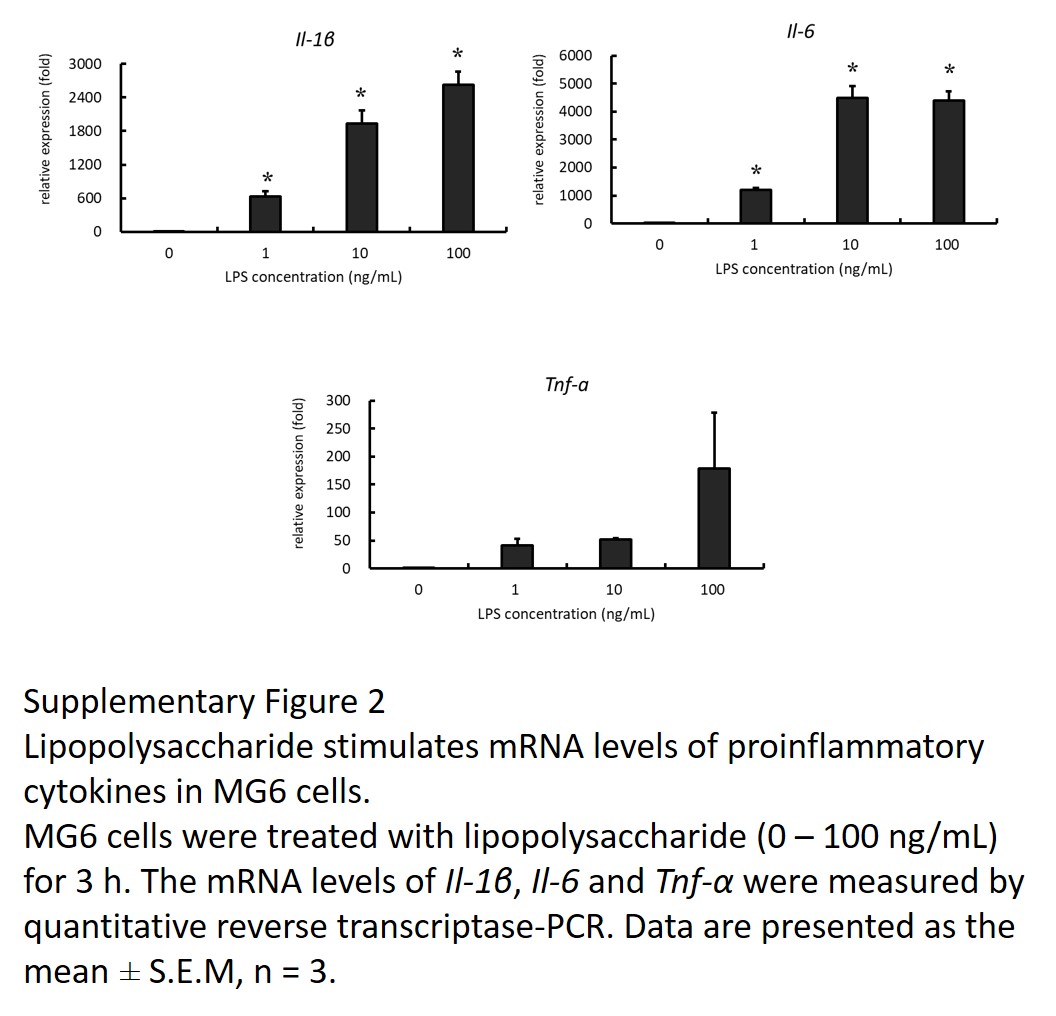

Supplement: Supplementary file 1 [file ijms-20-02317-s001.zip › Sup figures/Sup Fig 2.JPG]

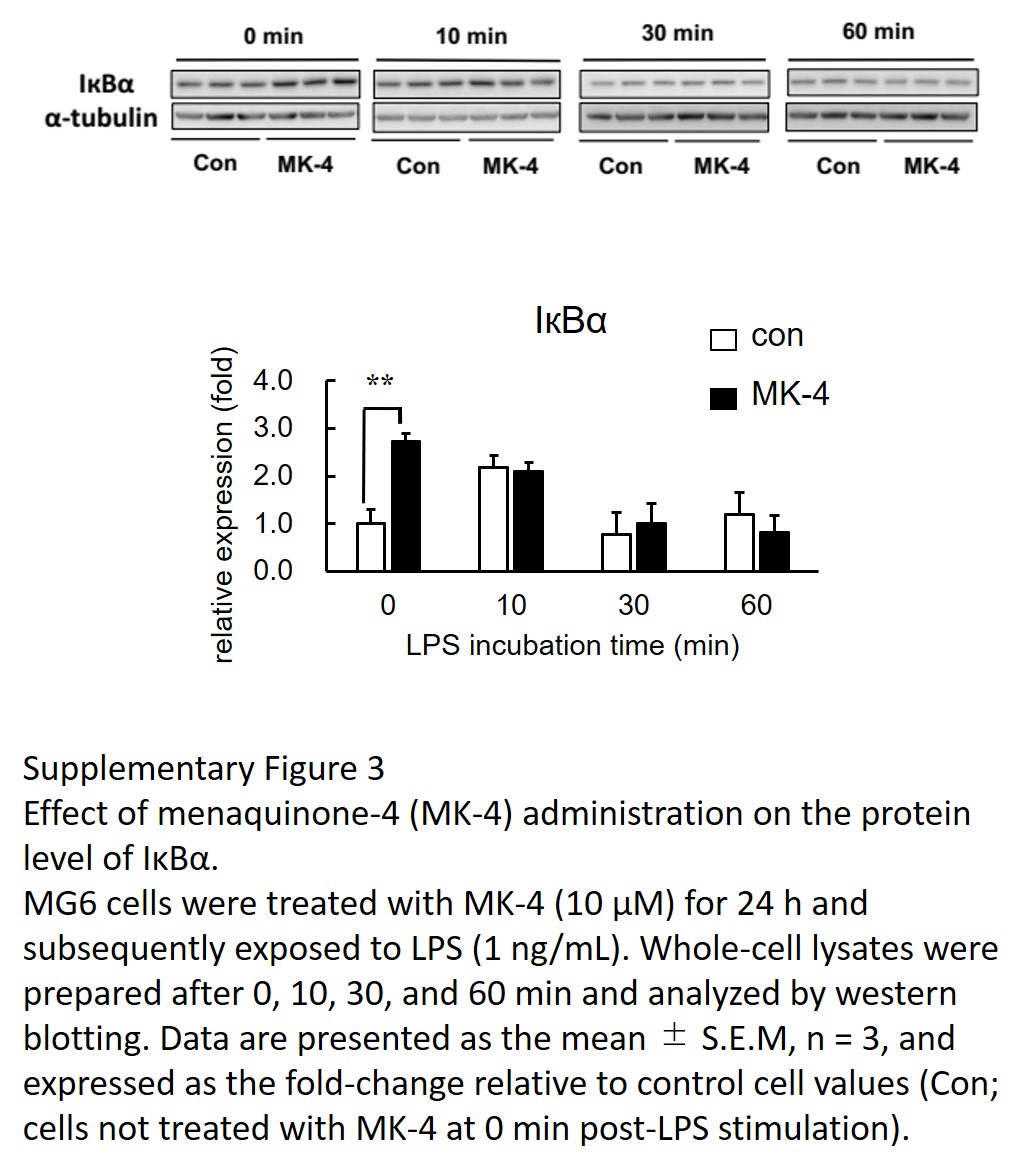

Supplement: Supplementary file 1 [file ijms-20-02317-s001.zip › Sup figures/Sup Fig 3.JPG]
